# Preserving Care Delivery in Hard-to-Serve Regions: A Case Study of a Population Health System in the Swiss Lower Engadin

**DOI:** 10.5334/ijic.3353

**Published:** 2018-07-03

**Authors:** Matthias Mitterlechner, Céline Hollfelder, Joachim Koppenberg

**Affiliations:** 1 University of St. Gallen, Healthcare Management, Dufourstrasse 40a, CH-9000 St. Gallen, CH; 2 University of St. Gallen, Institute for Systemic Management and Public Governance, Dufourstrasse 40a, CH-9000 St. Gallen, CH; 3 Center da sandà Engiadina Bassa and Department of Anaesthesiology, Pain Medicine, and Emergency Medicine, Via da l’Ospidal, CH-7550 Scuol, CH

**Keywords:** population health, integrated care, hard-to-serve regions

## Abstract

**Introduction:**

Many countries report difficulties in preserving access to care in rural areas. This paper examines how hard-to-serve regions sustain care provision by transforming service delivery into population health systems.

**Theory and methods:**

The paper builds on theory on care delivery in hard-to-serve regions. It presents a qualitative case study from the Lower Engadin, a rural high mountain valley in the Swiss Alps. Data sources include semi-structured interviews, participant observations, and documents. Data are analysed using recent conceptual research on population health systems.

**Results:**

The case study illustrates how politicians and providers in the Lower Engadin resolved a care crisis and preserved access to care by forming a population health system. The system is organised around the Healthcare Centre Lower Engadin. Citizen-centred interventions target an aging population and include health promotion and prevention programs as well as case management based on an ambulatory-before-inpatient care strategy.

**Conclusion:**

Hard-to-serve regions like the Lower Engadin preserve access to care by reorganising service delivery towards population health systems. The paper contributes to research on population health systems and care provision in rural areas.

## Introduction

Healthcare systems around the world are challenged not only by demographic aging and a growing number of patients with complex needs, but also by regional disparities in care provision [1,2,3,4,5]. Especially rural regions experience growing difficulties in sustaining access to affordable, high quality care [2]. To maintain care in these “hard-to-serve” [3] regions, research has recommended various strategies like the training of additional professionals, settlement bonuses, or regulation restricting the choice of practice location [6,7,8,9]. These strategies aim to address the problem by increasing the number of professionals. Other scholars view supply shortages not only as a quantitative problem, but also as a question of how care is delivered, arguing for qualitative organisational reforms. Knieps et al. [4] suggest that supply shortages should be addressed by questioning the traditional delivery system, and by developing new organisational models like population health systems, which “can make a decisive contribution to optimising care processes and preserving local care also in the future” (p.9, our translation). However, the concrete design and implementation of such reforms is an open question. Alderwick et al. [5] note that “previous attempts to prioritise population health have met with partial success at best,” cautioning that the challenges involved should not be underestimated (p. 23).

Against this background, this paper explores how hard-to-serve regions preserve access to care by reorganising service delivery and forming population health systems. To this end, it presents a case study describing the organisation, activities, formation, and impact of a population health system in the Lower Engadin, a rural high mountain valley in the Swiss Alps. Similar to other rural areas, the Lower Engadin faces delivery challenges like long travel distances, limits to economies of scale due to a small population size, and constrained access to resources like information and talent [2]. The case study shows how the region has coped with these challenges by transforming fragmented service delivery into a population health system spanning multiple providers and sectors. The system is organised around the Healthcare Centre Lower Engadin and considered as one of the pioneering population health systems in Switzerland [10,11,12].

This case study results from ongoing research in which we study leadership and governance questions in collaboration with integrated health and social care networks in Switzerland, drawing partly on data we used in our own previous work [13]. Our fieldwork in the Lower Engadin started in September 2011 and ended in November 2015. In this time, we collected longitudinal data including 35 semi-structured interviews, 96 participant observations, and 46 archival records. In our data analysis for this case study, we drew on recent conceptual research on population health systems [14,15]. With this paper, we wish to contribute to debates on care delivery in hard-to-serve regions and population health systems more generally [1,2,3,4,5,6,7,8,9,14,15].

The paper is structured as follows. First, we will provide conceptual background on population health systems. Second, we will describe the organisation and activities of the population health system in the Lower Engadin. Third, we will describe the formation of the system, outlining the starting conditions and implementation process. Fourth, we will report on intermediate outcomes. In conclusion, we will discuss insights for other hard-to-serve regions and contributions to the literature.

## Conceptual background on population health systems

The term “population health system” is increasingly mentioned as a strategy for realising high quality, effective, and affordable health systems [1,4,5,14,15,16]. While several definitions exist [14], population health systems can be understood as systems addressing the health needs and well-being of a specified population by integrating services across healthcare, prevention, social care, and welfare [1,15]. Spanning multiple sectors, they reach beyond the integration of healthcare to manage specific diseases, focusing on the broader determinants of health within a geographic area, including healthy behaviour, education, housing, or the environment [1,14,15]. Considering the broader determinants of health, they require collaboration across multiple providers from several sectors, local authorities, patients, and the public [5]. Examples are Jönköping County Council in Sweden [17], Gesundes Kinzigtal in Germany [18], Bidasoa Integrated Health Organisation in Spain [19], or accountable care organisations in the US [e.g. 20].

According to Struijs et al. [15], a population health system is *organised* around specific structures including legal form, governance, leadership, strategy, incentives and contractual arrangements. The *activities* of the organisation can be described based on six steps: (1) identification of a target population, (2) triple aim assessment, (3) risk stratification, (4) citizen-centred services, (5) impact evaluation, and (6) quality improvement [15]. Research also suggests that the *formation* of a population health system is influenced by contextual factors. Frequently mentioned challenges and facilitators are legislation, payment systems, data sharing, and provider readiness for change [1,14,15]. The *impact* of a population health system should be assessed according to the triple aim of health outcome, quality including accessibility of care, and costs of care [15]. The three goals are interdependent, which means that changes pursuing any one goal can affect the other two [16]. Due to time lags among change effects, Struijs et al. [15] also call for an assessment of how population health systems develop over time.

In the following, we will draw on these concepts to describe the population health system in the Lower Engadin, exploring its organisation, activities, formation process and impact.

## A population health system in the Swiss Alps

**Figure 1:**
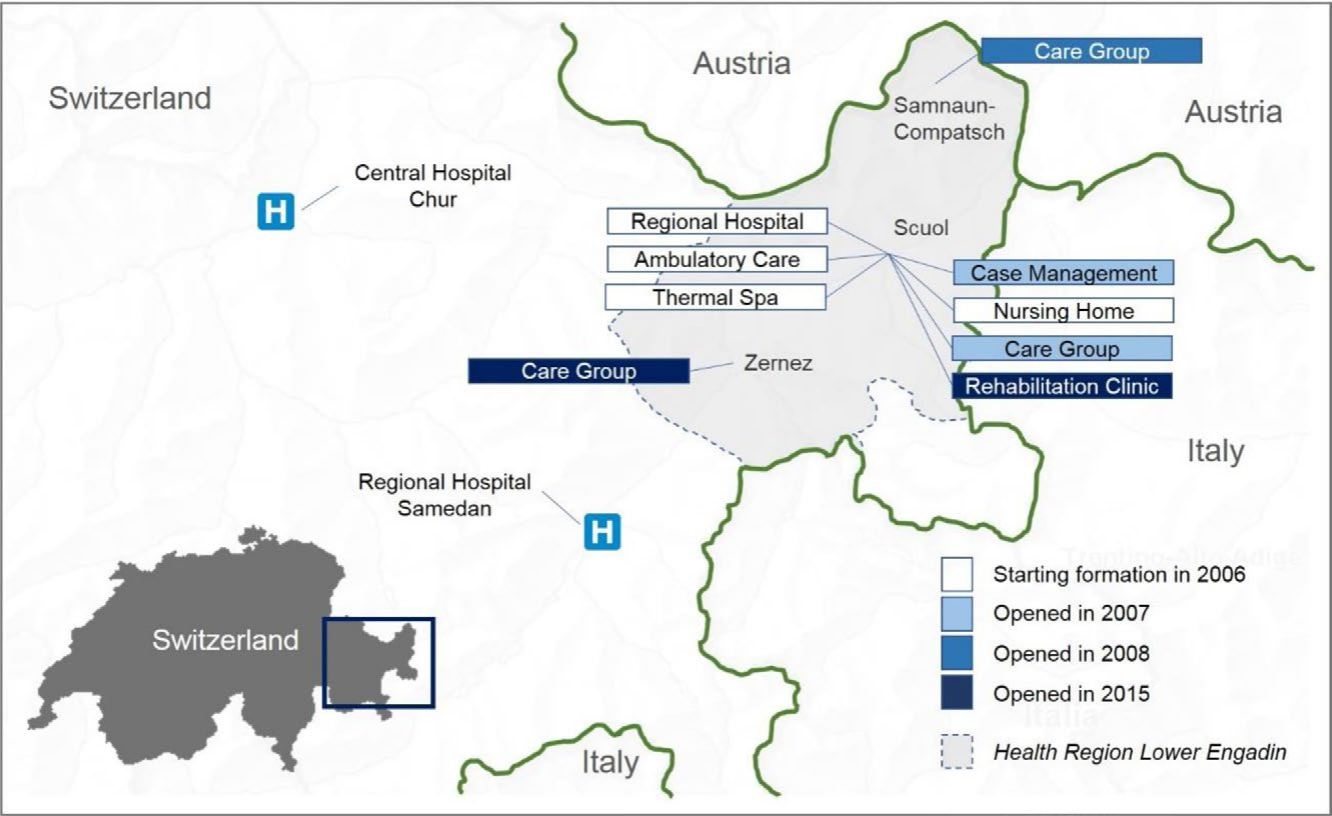
A population-oriented health system in the Swiss Alps.

Located in the canton Grisons, the Lower Engadin is an alpine high valley spanning roughly 1,000 km^2^ at Switzerland’s south-eastern border (see Figure 1). The main town of the region is Scuol, Switzerland’s most extended municipality with a size of 438 km^2^. The Lower Engadin is blessed with beautiful nature and rich cultural heritage, economically depending on tourism and associated sectors like commerce and construction. It is governed by a “Regional Council” composed of the mayors of the municipalities forming the region.

The permanent population counts roughly 8,000 inhabitants. It is both aging and older compared to the rest of Switzerland. In addition, the Lower Engadin is an attractive holiday destination for tourists generating almost 1 million overnight stays per year with peaks in summer (hiking, biking) and winter (alpine and cross-country skiing). The tourists originate mainly from Switzerland (90%) and other European countries (10%). Many tourists select the Lower Engadin as their destination not least because they can resort to high quality healthcare if needed.

Car transport from Scuol to the next regional hospital located in Samedan in the Upper Engadin takes roughly 1 hour. Transport to the next central hospital located in Chur, capital of Canton Grisons, takes 2 hours by car and 0.5–1 hour by air, although helicopter transport is possible only with favourable weather conditions. Ground transport to Chur is further complicated by mountain passes regularly closed in winter due to their altitude of up to 2,400 m.

This remote geographical location entails special challenges for providers to preserve high quality and affordable care. It makes it harder for them to access critical resources like information, services (e.g. second opinions) and qualified talent. It also limits economies of scale due to a small pool of regional patients. Moreover, providers have to supply extra capacities for the touristic peak season and find ways of utilising these resources during the low season.

To maintain access to care in this context, providers and regional politicians have reorganised service delivery and formed one of Switzerland’s pioneering population health systems.

### Population health organisation

**Figure 2:**
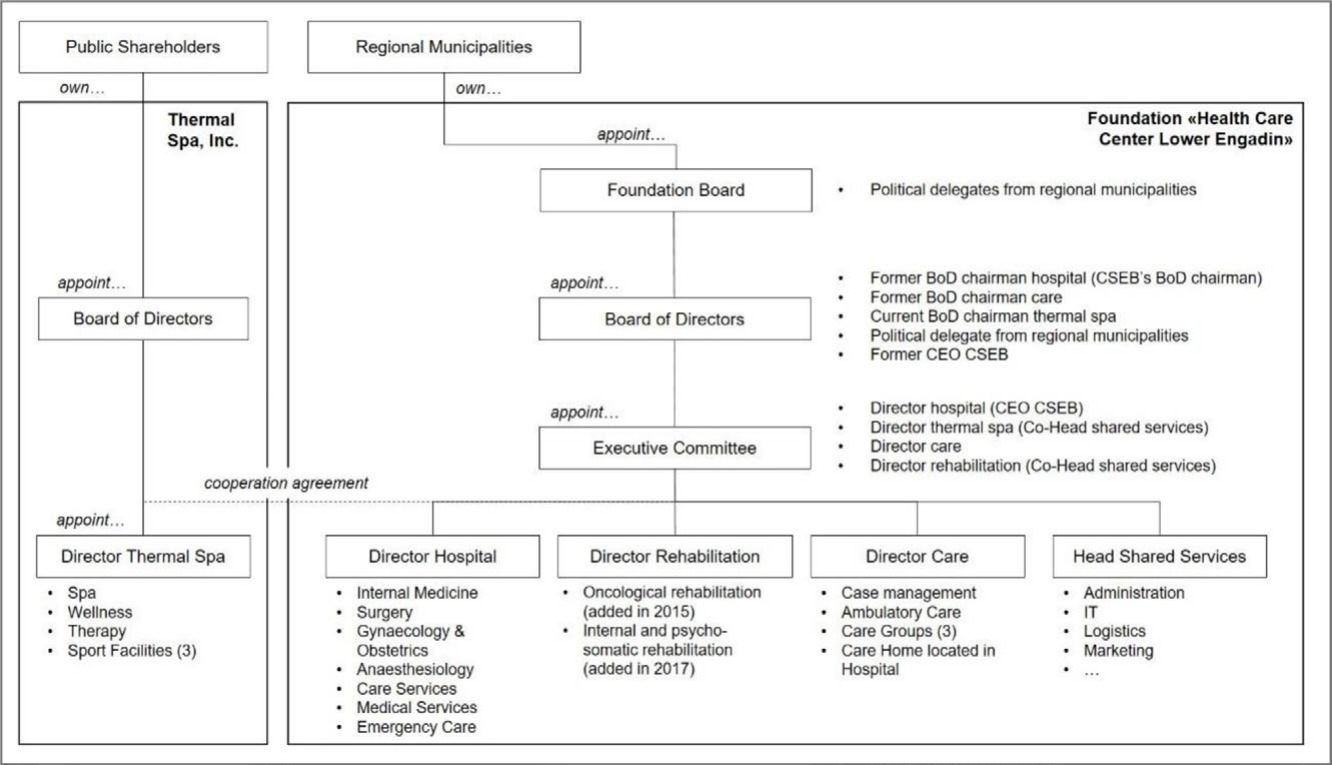
CSEB’s organisation as of January 2018.

The population health system is organised around the Healthcare Center Lower Engadin (Center da sandà Engiadina Bassa/CSEB), a foundation owned by the regional municipalities. As per January 1, 2007, politicians and service providers created CSEB from a merger between the regional hospital and ambulatory care organisation. At the same time, CSEB formed a strategic alliance with the regional thermal spa. Figure 2 shows CSEB’s organisation as of January 2018.

Mandated by the Regional Council with ensuring care provision for the Lower Engadin, CSEB orchestrates a regional network of provider organisations to deliver its services (see Table 2). Public network members include schools and social welfare organisations like the guardianship office. Private network members include a nursing home, a social services provider for elderly people, a dementia association, caring relatives and volunteers, sport clubs, hotels, restaurants, businesses, medical consultants and roughly ten general practitioner (GP) practices. The canton Grisons neither owns nor operates any providers in the Lower Engadin. While some of these network relations are governed by contractual provisions (those between CSEB and the thermal spa and medical consultants), most rely on trust and reputation.

**Table 1:** Demographic aging and rising demand for inpatient and ambulatory care.

	2005	2010	2015	2020	2025	2030

Population	7,600	7,650	7,700	7,750	7,800	7,800
Population aged 80 +	403	434	457	483	506	530
In % of total population	5.3%	5.7%	5.9%	6.2%	6.5%	6.8%

Demand for long-term inpatient care among 80 + in %	26.1%	26.3%	26.5%	26.7%	26.9%	27.0%
Demand for long-term inpatient care (number of clients)	105	114	121	129	136	143
Available spaces (as of 2007)	94	95	95	95	95	95
Long-term inpatient care shortage (as of 2007)	–11	–19	–26	–34	–41	–48

Demand for ambulatory care among 80 + in %	24.8%	25.0%	25.2%	25.4%	25.6%	25.7%
Demand for ambulatory care (number of clients)	100	109	115	123	130	136

**Table 2:** Key services and segments of the Lower Engadin’s population health system.

Services	Population segment	Service offerings (in italics: new services added since CSEB’s foundation in 2007)	Network members

*Health promotion and prevention*	*Children and adolescents*	*Advice on movement and nutrition, organisation of leisure time and addiction prevention for adolescents*.	*Schools, sports clubs*
	*Middle-aged adults*	*Health promotion and prevention in the middle years and at the work place: Coping with stress, dealing with new media*.	*Businesses*
	*Elderly people*	*Future workshops, preventive home visits, health checks, housing counselling*.	*Social services*

Ambulatory primary care	Whole target population	Mobile rescue service in three locations. Ambulatory emergency care in regional hospital.	
Ambulatory specialised care		Medical consultancy in *ophthalmology, dermatology*, gastroenterology, *neuropediatrics, oncology, otolaryngology*, paediatrics, *psychiatry*, radiology, and *wound management*.	*Private medical consultants*

Inpatient primary care	Whole target population	Surgery (traumatology, orthopaedics, visceral surgery, manual medicine); internal medicine (cardiology, angiology, pulmonology, sports medicine, palliative care); obstetrics and gynaecology; anaesthesia (pain therapy, transfusion medicine, emergency medicine); complementary medicine.

*Rehabilitation*	*Whole target population*	*Inpatient oncological, internal and psycho-somatic rehabilitation in regional hospital and hotels. Ambulatory physiotherapy and physical medicine in the thermal spa*.	*Hotels, private medical consultants*

Ambulatory home care and inpatient long-term care	Elderly people	Ambulatory home care: Help and care in crisis situations, support and relief of caring relatives, post-acute health and nursing services, housekeeping and meal services.	
		Inpatient long-term care in the regional hospital and three decentralised care groups in collaboration with a regional nursing home.	Private nursing home
		*Dementia care in long-term facilities*.	*Dementia association*

*Case management*	*Elderly people*	*Central contact point for all questions related to aging and care in the region. Advice for patients and their relatives with complex care needs*.	*Dementia association, social welfare, private GPs, private volunteers*
		*Referral of temporary inpatient care services; implementing health promotion and prevention programs; maintaining a pool of regional volunteers for providing ambulatory care and social services*.	

*Health tourism*	*Tourists*	*In collaboration with the regional tourism industry, CSEB offers a range of health tourism products in order to add positive contribution margins to its operations. The offering includes health promotion and prevention (e.g. health checks in the regional hospital, workshops and seminars); gluten- and lactose-free holidays in collaboration with regional hotels, restaurants and businesses; temporary inpatient care for holiday guests with care needs; wheelchair accessible holidays; and lectures and regional health experiences*.	*Accomodation providers, restaurants, businesses, private medical consultants*

*Integrated shared services*	*n/a*	*CSEB and the thermal spa pool their shared services including IT, marketing, finance, HR, and logistics to increase the efficiency and quality of these services*.

Day-to-day coordination in this system involving multiple organisations, professions, and work practices occurs by means of electronic and face-to-face communication. Electronic communication currently focuses on e-mail. An integrated IT infrastructure for sharing patient and other information across the system is under construction. Face-to-face communication is required for coordinating clinical activities like wound management, fall prevention, hygiene, pain treatment, palliative care or patient safety. CSEB coordinates these activities by hosting topic-related quality circles composed of delegates from involved professions and providers. The delegates meet face-to-face, develop treatment guidelines, and implement the guidelines within their respective organisational units.

The organisations participating in this system are funded privately (e.g. commerce) or based on national and cantonal regulations (healthcare and social services providers). For example, the regional hospital is financed based on Swiss DRG, the nationwide fee-per-case system. Possible deficits are carried by the regional municipalities owning CSEB and thus the hospital. An exception is CSEB’s case management office, which receives direct funds from the regional municipalities due to a lack of adequate national or cantonal reimbursements for this coordination service. The Swiss tariff system is generally tailored to individual services of providers and lacks incentives for the coordinated treatment across providers [21].

The regional population is insured via Switzerland’s statutory health insurance (SHI) scheme. SHI is universal and mandatory for all Swiss residents and offered by about 60 competing insurance companies. In 2017, the annual premium for Lower Engadin adults averaged roughly CHF 4,500 (vs. roughly CHF 5,400 for entire Switzerland). SHI covers most primary care services including those of hospitals, GPs, specialists, physiotherapists, rehabilitation clinics, and ambulatory care. Insurance companies are obliged to offer annual deductibles ranging from CHF 300 to CHF 2,500, with higher deductibles lowering premiums. Above deductibles, insured persons also pay 10% coinsurance for all services with a cap of CHF 700 in a given year and CHF 15 for acute hospital treatments per inpatient day. On average, one third of Swiss healthcare spending comes out-of-pocket from such co-payments, deductibles and other private payments. In addition to SHI, residents may also purchase voluntary health insurance (VHI), which offers extra services and amenities like higher levels of hospital accommodation (see [22] for more information).

### Population health activities

In 2007, the Regional Council mandated CSEB with ensuring care provision in the Lower Engadin. The *target population* (activity 1) primarily comprises permanent citizens. Due to the importance of tourism, it also includes the guests visiting the region. Figure 1 depicts the geographical scope of the so-called “Health Region Lower Engadin.”

To *assess permanent citizens’ care needs* (activity 2), CSEB has used demographic and epidemiological data (see Table 1). The data indicate an aging population with growing demand for inpatient long-term and ambulatory care services. Given these needs, CSEB aims to create a system that reconciles the growing demand for care with the limited financial means of the Lower Engadin. “We aim to account for the needs of care recipients, a meaningful and equitable social development, and the economic and financial means of the public” (CSEB, strategic plan, 2007).

To do so, CSEB collaborates with network members to provide a range of *citizen-centred services, segmenting the target population into age groups* (activities 3 and 4, see Table 2). The offering spans the whole spectrum of a population health system, from obstetrics to palliative care. The following service lines warrant a few additional remarks:

- *Health promotion and prevention:* Mandated by the Regional Council, CSEB is responsible for designing health promotion and prevention programs for four age groups: (1) movement and nutrition for children and adolescents; (2) organisation of leisure time and addiction prevention for adolescents; (3) health promotion and prevention for middle aged people; (4) health and fall prevention for elderly people. Given demographic aging, CSEB’s activities have recently focused on the fourth segment. Since 2013, CSEB has reached 535 elderly people in workshops across the region, reflecting with them and their relatives on the meaning of healthy aging and strategic priorities until 2022. Additional activities for this segment include regular health checks, home visits, and housing counselling to prevent falls.
- *Ambulatory home care and inpatient long-term care:* These services are consolidated in CSEB’s “Care” department (see Figure 2). To cope with growing aging-related care demand, CSEB aims to support patients and clients with tailor-made services as long as possible at their homes rather than in inpatient long-term institutions. Case management centrally handles the coordination of these services.
- *Case management:* Headed by a nurse, the case management office is the single point of entry for all questions related to aging and care. It advises relatives and patients with complex needs requiring several services across CSEB and network members. In line with the ambulatory-before-inpatient care strategy, it also provides temporary inpatient care beds for relieving caring relatives, designs and implements health promotion and prevention programs, and develops a pool of volunteers providing ambulatory care and social services.
- *Health tourism:* In collaboration with accommodation providers, restaurants, businesses, and medical consultants, CSEB offers health tourism services including rehabilitation. With these services, CSEB expects to attract new visitors using idle capacity during the off-peak season and thus adding positive contribution margins to co-finance the regional population health system.

To *ensure the quality* of these services (activity 6), CSEB has created two platforms. First, the “Care Management Board” ensures the quality of all citizen-centred services, aiming to optimise patient processes and information flows across CSEB and network members. Second, the “Health Tourism Board” is responsible for monitoring the performance, launching new products, and improving the quality of all health tourism services. Both boards are chaired by senior managers from CSEB and involve regional network members via network-wide meetings, bilateral talks, and training programs. These events are occasions not only for ensuring service quality, but also for governing the system by building trust and reputation among network members.

Before assessing the preliminary *impact* of the population health system (activity 5), we will now describe the formation of the system.

### Formation process

Around the turn of the millennium, the care system in the Lower Engadin was far from what it looks like today. Service delivery among providers was fragmented and mutual recriminations in case of problems were daily fare. This section will describe how regional actors leveraged a regional crisis to transform fragmented service delivery into an integrated population health system, dividing the process into three periods.

#### Period 1: A region in crisis (2003–2006)

Between 2003 and 2006, the Lower Engadin’s care providers were challenged by existential internal problems and an escalating shortage of long-term inpatient care.

The *hospital* was in an existential crisis. Due to budget problems, the cantonal health department, which regulated, licensed and co-financed healthcare in the canton, reviewed the services of the hospital. They proposed to close the orthopaedics department and replace obstetrics with ambulatory delivery nurses. Hospital executives were concerned that this plan would erode the economic viability of their organisation. The *ambulatory care organisation* was entangled in an escalating long-term care crisis. Since the regional nursing home increasingly lacked the capacity to meet the growing demand for inpatient care, elderly citizens started rotating between acute and ambulatory care until they could be accommodated in the nursing home. This situation created discontent, prompting citizens to write complaint letters to the Regional Council. The *thermal spa* faced competition from facilities in Switzerland and abroad. Faced with declining margins, they looked for new ways of diversifying revenues and reducing production costs.

Alarmed by these problems, the Regional Council appointed a management team to find solutions. They composed the team of the CEOs and BoD chairmen of the affected organisations plus a “healthcare delegate” from their own ranks. The team quickly established trust based on productive work relationships among equals. “At the strategic level, we were fortunate to have people who did not have to distinguish themselves. Nobody had to be the boss or the king” (management team member). After they had travelled to study a structurally similar region, they submitted an innovative proposal.

The management team suggested merging the hospital and ambulatory care organisation into a foundation called CSEB, which would form an alliance with the thermal spa. This would create opportunities for jointly developing new products, sharing expertise, coordinating patient activities, and realising cost synergies by centralising shared services. Some politicians were concerned that the merger would entail an undesirable blend of financial flows and cross-subsidies for instance between the hospital and ambulatory care organisation. To address these concerns, the management team proposed that all parties would remain financially independent based on transparent financial reporting and continue carrying their own entrepreneurial risks.

The Regional Council accepted the proposal. CSEB was launched on January 1, 2007.

#### Period 2: CSEB’s pilot phase (2007–2008)

Supported by the Regional Council, the management team took next steps. To calm high expectations from politicians and employees, they officially communicated this period as a “pilot phase,” emphasising CSEB’s experimental character.

In a first step, they defined CSEB’s governance. In workshops, they delineated competencies across hierarchical levels, specifying roles and responsibilities of the Foundation Board, Board of Directors, and Executive Committee. This governance structure would prevent politicians’ interference in CSEB’s operational business, as it had previously sometimes been the case in the hospital.

In a second step, they coordinated support activities. Centralising finance, HR, IT, marketing, logistics, laundry, food and beverage, and real estate in a shared services department, they aimed at realising cost and investment synergies.

In a third step, they addressed the inpatient care shortage, mandated by the Regional Council to preserve care in the Lower Engadin. Coordinating with regional stakeholders including politicians, citizens, professionals, and social service providers, they created an “ambulatory-before-inpatient” care strategy. To implement the strategy, they proposed to establish a case management unit, which would coordinate care and develop health promotion and prevention programs for elderly citizens. Due to a lack of national or cantonal reimbursements for care coordination, they proposed to finance case management with funds from the regional municipalities, calculating that in the end these costs would be lower than investments in long-term care capacities. In addition, to resolve the immediate shortage, they suggested approving 15 inpatient care beds in the nursing home, and building three decentral “care groups” under CSEB’s leadership. The care groups would be rented apartments and provide new space for 23 elderly people. The strategy was approved by the Regional Council and calmed the long-term care crisis.

#### Period 3: Coordinating patient-centred activities and health tourism (from 2009)

In this period, the management team started coordinating patient-centred activities, establishing a trans-sectoral “Care Management Board” aiming to improve the quality of citizen-centred services. Analysing CSEB’s still fragmented patient processes, the Care Management Board developed a shared protocol for discharging patients from the hospital, matching patient needs with information requirements and responsibilities. Patients with complex needs would be referred to case management, which would assume responsibility for defining post-acute care options and pathways in dialogue with patients, their families, and CSEB’s operations.

In the wake of their analyses, the Care Management Board realised that complex patients would require the support from additional regional social services organisations, e.g. from the guardianship office or the dementia association. To establish trust and coordinate service activities, they established ties to these providers, regularly inviting them to network-wide meetings, bilateral talks, and training programs.

Moreover, the management team launched an initiative to create new health tourism services in collaboration with the regional tourism association and providers including hotels, restaurants, and businesses. They aimed to attract new extra-regional clients and patients co-financing CSEB’s and network members’ fixed costs during the low season.

As an emergent system, CSEB’s population health system continues to evolve. Two priorities stand out. On one hand, CSEB’s management team aims to strengthen ties to network members. For instance, they are in conversations with people from the nursing home, reflecting on how they could optimise the provision of long-term care.

On the other hand, they focus on the digitalisation of the value chain. They invest in online research databases, videoconference systems, tele-radiology, real time transmission of ECG data to mobile devices, and online stroke therapy in collaboration with specialists from a large central hospital (St. Gallen). They also upgrade CSEB’s IT system to share information across the system, aiming to keep pace with medical progress and compensate for their remote geographical location.

### Impact assessment

This section will report on how the population health system has affected the accessibility, affordability, cost, and quality of care. Population health data are not (yet) available; more longitudinal and comparative research would be needed.

First, the system has helped the hospital, ambulatory care organisation, and thermal spa overcome their problems. Given the regional care crisis and debates about closing the hospital, it is not exaggerated to argue that the formation of the system preserved the existence of these providers and hence *access to care* in the Lower Engadin.

**Figure 3:**
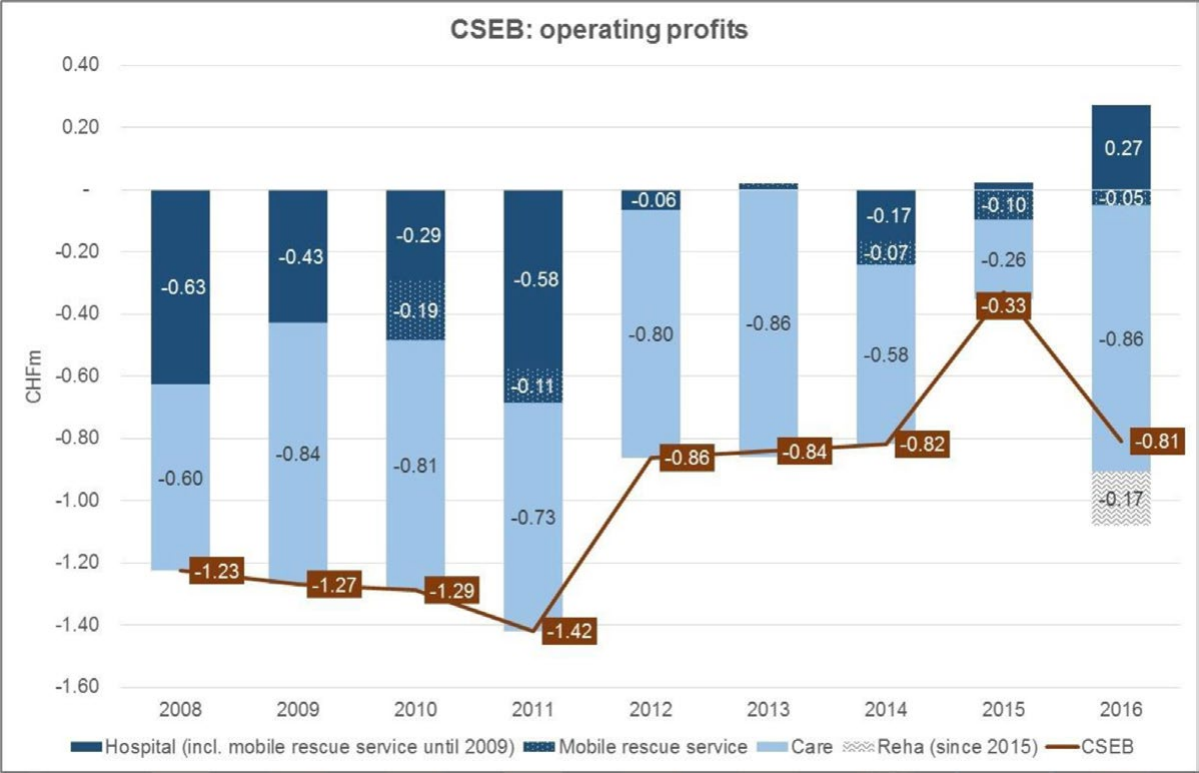
CSEB’s operating profits 2008–2016 (Source: CSEB Controlling).

Second, data suggest that the system has helped the region *sustain an affordable care provision*. Figure 3 shows that CSEB’s operating losses at the expense of the municipalities dropped by a third between 2008 and 2016, a development mainly driven by the hospital.

After years of chronic losses, the hospital practically broke even between 2012 and 2016, showing small profits in 2013 and 2016. A first explanation are economies of scale resulting from pooled shared services. A second explanation are efficiency gains due to process improvements. Between 2008 and 2016, the hospital reduced patients’ average length of stay (LoS) from 7.1 to 6.4 days, which contributed to declining losses under Switzerland’s fee-per-case remuneration system (“Swiss DRG”) introduced in 2012. The reduction of patients’ LoS was enabled by an internal process improvement program and CSEB’s ambulatory-before-inpatient care strategy, which included an optimised hospital discharge process and case management. The number of case management clients increased from 37 in 2012 to 91 in 2016 (2013: 103; 2014: 78; 2015: 123). A final explanation are increasing patient numbers driven by an expanding high quality service range. Between 2008 and 2016, the number of inpatient and ambulatory cases climbed from 6,683 to 7,659, an increase by 14%. Under Swiss DRG, revenues, contribution margins, and profits increase with falling LoS and growing patient numbers.

**Figure 4:**
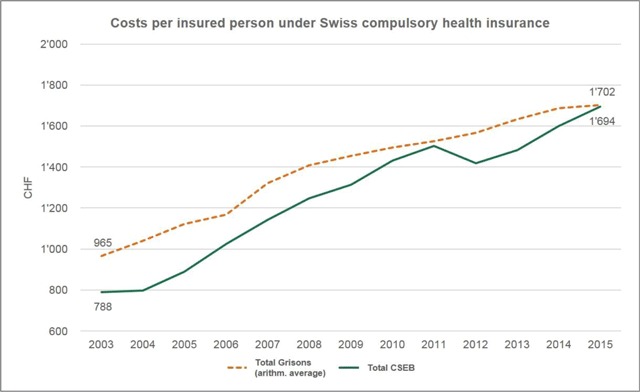
Healthcare costs per insured person (data are standardised according to sex and age). Healthcare costs include ambulatory and inpatient costs for care and the hospital as well as physical therapy, reflecting CSEB’s services (Source: Swiss Health Observatory, Health Department Canton Grisons).

Third, data also suggest that the system has helped the Lower Engadin deliver care at lower costs per capita than the cantonal average. To control for cantonal differences in healthcare policy and legislation, Figure 4 compares the costs per insured person under Switzerland’s statutory health insurance scheme (SHI) for the Lower Engadin with those for the canton Grisons. It indicates that CSEB’s costs per insured person have been lower compared to those of the cantonal average. However, it also shows that CSEB’s costs have recently outgrown the benchmark, almost equalising it in 2015. This above-average growth can be explained by increasing patient numbers in the hospital and the expansion of CSEB’s service range as shown in Table 2.

**Figure 5:**
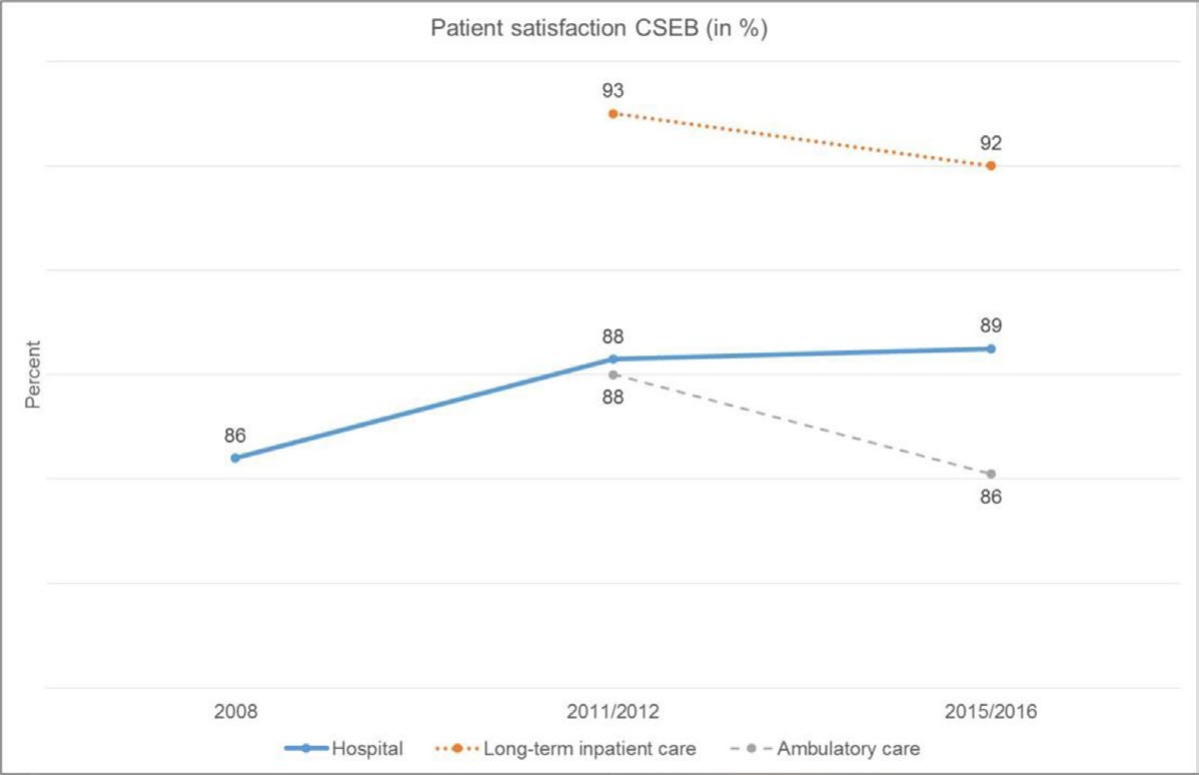
Patient satisfaction with CSEB’s services (Source: CSEB Controlling).

Fourth, the system has facilitated *not only affordable, but also high quality* care. Figure 5 shows that patient satisfaction rates with CSEB’s services are at constantly high levels. The share of patients or clients very satisfied with hospital and ambulatory care services adds up to roughly 86–89%. The share is even higher for inpatient care clients, moving around 92–93%. Hospital re-admission and re-surgery ratios mirror CSEB’s high quality of care. They are either below or in line with the national benchmark (Source: ANQ/Swiss national association for quality development in hospitals and clinics, 2015).

Looking at these numbers, one of CSEB’s BoD members notes, “I think that the regional population trusts CSEB. They consider us as an important partner and use our services. They don’t have the feeling that services are always better in other places.” This result is remarkable given citizens’ frustrations with care provision at the turn of the millennium. A further evidence of citizens’ trust in CSEB was the acceptance of a recent referendum on building a new CHF 16.7 million operating room in the hospital. The investment was supported by 92% of the population.

Finally, the system has had important *economic and cultural effects* on the region. Economically, CSEB has become the largest employer in the region, adding 60 full-time jobs including eight apprentice positions since 2007. Culturally, CSEB’s formation has enhanced social cohesion. Noted a Regional Council member, “I also believe that CSEB contributes a lot that the region grows together even stronger than before.”

## Discussion

Policy makers in many countries face the question of how they can preserve access to care in remote areas [2,6,7,8,9]. Although research recommends reorganising service delivery into population health systems, there is still much uncertainty about the concrete design and implementation of such reforms [5]. This case study suggests important insights into the organisation, activities, formation, and impact of this transformation.

First, regarding *organisation*, the case suggests that rural regions can benefit from building an “integrator organisation” responsible for system coordination and resource allocation [14,16]. In the Lower Engadin, CSEB is charged with coordinating activities across its own organisation and a regional provider network. This accords with previous research viewing the existence of an integrator as a precondition of an effective population health system [16]. In this case, the integrator is a publicly owned foundation coordinating services across a network of other public and private providers. This raises the question of whether such a system could be realised by private actors alone. While a private solution is theoretically conceivable, CSEB’s ongoing deficits indicate sustained challenges in delivering care in rural regions in ways that are profitable and attractive to private providers. In general, therefore, this case seems to suggest that public private partnerships coordinated by a public integrator organisation constitute a feasible way of reforming care delivery in rural regions.

Second, the case suggests valuable insights into useful population health *activities*. Creating a system around the needs of the target population, CSEB has stratified citizens into age groups, using basic demographic data. This segmentation has been helpful as a first step, although it is less elaborate than discussed in literatures calling for more elaborate triple aim assessments [15]. Going forward, the development of an integrated IT infrastructure and data warehouse would however allow for more differentiated risk stratifications and citizens-centred interventions.

Faced with demographic aging, CSEB tailors regional services to the growing demand for ambulatory and long-term inpatient care, investing in health promotion and prevention, and operating a case management unit. The business case suggests that the underlying ambulatory-before-inpatient care strategy eventually leads to better and cheaper care than additional regional investments in inpatient care capacities. Other rural regions challenged by demographic aging may consider a similar strategy.

The case also indicates that rural regions can enhance the affordability of care by centralising support activities and developing new revenue sources. By pooling shared services, they improve expertise and realise economies of scale. Tapping into new revenue sources (like health tourism) helps utilise idle resources and finance fixed costs.

Third, the case study sheds light on important facilitators and challenges enabling and constraining the *formation* of population health systems in rural regions [15].

A key facilitator is *leadership*. The formation of the system in the Lower Engadin has been driven by a highly committed management team collaborating based on trust and relationships among equals. This finding echoes previous research similarly emphasising the central role of leadership for building population health systems [5,19].

The formation of a population health system also relies on a *favourable political context*. In this case, the context has been marked by a productive mix between pressure and support. On one hand, the cantonal service portfolio review and regional citizens’ complaints exerted substantial pressures on the old system. On the other hand, regional politicians assumed a facilitative role, appointing a regional management team, and accompanying their work with a delegate. Moreover, from period 2, the relationship between the political and provider level has been regulated by a strict governance preventing political interference into the daily operations of the system. This finding also adds to earlier studies showing how population health systems rely on sound working relationships and explicit role clarifications among politicians and suppliers [17].

A further helpful practice is to *divide the formation into several steps*. Forming a population health system is a complex task involving many stakeholders and activities. This case suggests that it can be useful to divide the formation into several digestible work packages, which comprise the definition of a robust governance, pooling support activities, coordinating citizen-centred activities, and developing a regional provider network. Moreover, the case suggests that it can be useful to frame the formation or parts of it as a “pilot” to manage high stakeholder expectations regarding feasibility and speed.

A major challenge has been *data availability*. Due to a lack of an IT platform in the early periods of the formation, CSEB resorted to publicly available demographic data to form the system. However, the further development of the population health system will be facilitated by a data warehouse including better access to triple aim data [15].

Another big challenge has been the *funding of care coordination*. Similar to other countries, Switzerland’s fee-for-service system lacks incentives for coordinating patients across providers [5,15,17,18,19]. Actors in the Lower Engadin developed a creative solution, financing case management with regional funds. While such local solutions are helpful, payment reforms at the national level could help remove disincentives for collaboration and support citizen-centred care coordination in the Lower Engadin and other remote areas [21].

Finally, the *impact* analysis suggests positive effects of service delivery reforms like population health systems on the accessibility, affordability, and quality of care in rural regions [2,4]. However, the analysis also reveals that the formation of such systems does not necessarily lower the costs of care, pointing to trade-offs among triple aim targets [16]. In this case, quality improvements and higher utilisation rates were associated with relative cost increases. At the same time, the case indicates that forming population health systems can have important effects beyond the triple aim targets. In the Lower Engadin, the system has created substantial socio-economic benefits, adding jobs and strengthening the regional community. It will be important to monitor and reflexively manage these different outcomes and trade-offs in the future development of the system. For instance, the currently discussed national reforms to lower costs of care will require new interventions to preserve access to care also in a more austere financial environment [15].

With these insights, this paper contributes to research on population health systems and care delivery in rural regions. First, given the multiple challenges involved in population health systems, it partly supports and adds new insights into their organisation, activities, formation, and impact, as discussed above [1,5,14,15]. Second, it contributes to research on care delivery in hard-to-serve regions. Previous research in this field has tended to connect care shortages to a quantitative lack of resources, offering policy strategies aimed at supply increases like interventions in medical training, financial settlement incentives or laws regulating the choice of practice location [2,6,7,8,9]. This paper complements this line of research, showing how rural region may respond to care shortages not only with capacity increases, but also with forming population health systems. It thereby makes an important contribution to research calling for more profound qualitative service delivery reforms to sustain care in hard-to-serve regions [2,4].

## Conclusion

Many countries report difficulties in preserving access to care in remote regions. This paper explored how rural regions sustain affordable high quality care by transforming service delivery into population health systems. Presenting a case from the Swiss Lower Engadin, it improves our understanding of the organisation, activities, formation, and impact of such systems. In this way, it may inspire other hard-to-serve regions embarking on similar journeys.

## Author Informations

Matthias Mitterlechner is Assistant Professor of Healthcare Management at the University of St. Gallen, where he received his PhD in organisation studies. Before entering academia, he worked in healthcare and the financial services industry. His research focuses on leadership and governance in inter-organisational networks with an empirical emphasis on population health and integrated care.

Céline Hollfelder has a background in sociology and organisation studies. She conducts her research at the research program “HealthCare Excellence” at the University of St. Gallen, where she studies strategic change and multidisciplinary collaboration in healthcare organisations and networks.

Joachim Koppenberg is CEO of the Center da sandà Engiadina Bassa (Healthcare Centre Lower Engadin), where he is also Head Physician of the Department of Anaesthesiology, Pain Medicine, and Emergency Medicine. In his research, he focuses on emergency medicine, patient safety, and integrated care.
